# 

**Published:** 2011-02-25

**Authors:** F Popa

**Affiliations:** ‘Carol Davila’ University of Medicine and PharmacyRomania

The beginning of 2011 marks the 4th year of publishing of ‘Journal of Medicine and Life’, under the aegis of the prestigious ‘Carol Davila’ University Press, ‘Carol Davila’ University of Medicine and Pharmacy, Bucharest, becoming, presently, an important tool for publishing studies, research and reviews from different branches of medicine, both for the professors and doctors and for the students and PhD.

For ‘Journal of Medicine and Life’, the new year presupposes a change, a change which will improve the quality of the published material and the co–optation of new prestigious authors. The final purpose is the indexing of the journal in as many international databases as possible, an action which will bring a high degree of visibility to the medical research in Romania, as well as to the authors who publish articles in it. Starting with the first issue of this year (vol. 4, no. 1), Journal of Medicine and Life aligns to the peer–review criteria and methodology, used by the international publications.

I wish to thank the authors who have contributed and who are still bringing their contribution to the improvement of the journal, but also the board and the editorial team who have made the image of the journal known at a national and international level, by participating in different events and by indexing it in some prestigious international databases such as PubMed, Index Copernicus, EBSCO Publishing and ProQuest.

In order to be helpful for the ones who constantly bring their contribution to the process of raising the notoriety of the journal, we intend that each issue of the journal to have a section dedicated to the young researchers and PhDs who want to make their researches or studies known in the pages of Journal of Medicine and Life. Accordingly, we have created, starting with the first issue of 2011, a new category called ‘Young Researchers Area’. Moreover, we wish to offer the possibility to some of the world's medical and academic personalities to publish prestigious articles in the journal, by creating another section named ‘Guest editor’.

The journal remains a tool of promoting the visibility of the researchers engaged in doctoral and postdoctoral studies, as well as a tool for information dissemination through the financed research projects. So, once again, we open the pages of the journal with pleasure, to all the friends, professors in the universities of medicine and doctors in the hospitals in our country but also from abroad, to the researchers, students and PhDs who wish to facilitate the professional dialogue with the whole world, for the good of all those who are suffering. 

